# Mind Your Own Business

**Published:** 1900-06-30

**Authors:** 


### The Hospital

A Journal of

The Medical Sciences and Hospital Administration.

Vol. XXVIII.—No. 718.] Edited by Sir Henry Burdett, K.C.B., and Solomon C. Smith, M.D., M.R.C.P. [June 30, 1900.

### Mind Your Own Business.

To a medical practitioner, even more than to the generality of mankind, it is a matter of the first importance that he should "mind his own business," and this for two reasons. First, because he is a person to whom people run on all sorts of occasions and appeal in every kind of difficulty, and is therefore liable to become involved in all sorts of quarrels; and secondly, because his opinion is apt to be taken as meaning more than that of an ordinary person, far more in many cases than he himself ever intended it to mean. This is especially the case when he puts his pen to paper and indites what the public delights to regard as a "certificate." Medical men will do well to ponder carefully over the case of Dowling v. Dods, recently tried in the Queen's Bench Division of the High Court of Justice. In this case damages were claimed from the defendant, a medical practitioner, for a libel contained in a certificate addressed to the relieving officer, and stating among other things that the plaintiff was "of unsound mind." The Judge held that the occasion was privileged, but left certain questions as to malice and as to the truth of the statements contained in the libel, with the result that judgment was entered for the plaintiff for £100. Let us point out at once that the privilege in this case did not depend upon the fact that the defendant was a medical man.

The Lunacy Act of 1890 provides that every constable, relieving officer, and overseer of a parish, who has knowledge that a person within his district (who is not a pauper and not wandering at large, conditions otherwise provided for) is deemed to be a lunatic and not under proper care or control, shall give information thereof to a Justice of the Peace, and that certain prescribed steps shall be taken. In regard to this, according to the *Times* report, Mr. Justice Darling said that there could be no doubt that if the relieving officer had expressed the opinion that the plaintiff was insane the statement would have been privileged, and he added that "it was impossible to suppose that the relieving officer was intended to act only in cases which came under his own observation. The policy of the Act required that information should be supplied to him by members of the public who had seen the behaviour of supposed lunatics. He was, therefore, of opinion that the defendant had acted on a privileged occasion." Medical men are constantly asked for certificates in regard to persons of this class; indeed, in many districts the impression is commonly held that such a certificate is a necessary preliminary to the intervention of the relieving officer It is, then, very satisfactory to find it laid down by the judge that such a certificate, if properly given, is a privileged communication. It must be understood, however, that the giving of such a certificate is not a medical function. There is no statutory obligation laid upon any medical man to give such a certificate, nor will the withholding of it prevent the law being put in operation. The document is "in the nature of information given to the relieving officer," it may be given by anyone who knows the facts, and it is only a privileged communication so far as it is true and is given in good faith and without malice. Hence the importance of a medical man minding his own business, and not meddling in other people's concerns. It is, no doubt, very nice for these other people, when they want to be relieved of the care of a lunatic, to run round to a doctor, and induce him to write a "certificate" informing the relieving officer that So-and-so is a lunatic not under proper care or control; but it is not very nice for the doctor to find himself cast in damages for his intervention in what really was not his affair. No medical man who is in actual attendance upon a patient who becomes insane, and is left "not under proper care or control" need hesitate to sign a certificate to that effect, or, in other words, to become an informant, if it is necessary for the good of his patient that the relieving officer should intervene. The work comes before him in the ordinary way of duty, he has had ample opportunity of ascertaining for himself the facts of the case, his certificate is given *boná-fide*, and is without "malice" even in the legal sense. But when a medical man is called in merely to give a certificate, let him beware. Malice is an elastic term, and in view of the fact that the document asked for is "in the nature of an information," and by no means necessarily one to be given by a medical man, no man can be sure that the fact of his giving such a certificate largely on hearsay, especially if done for a fee, might not be turned against him. We feel sure that a judge would protect a medical man acting *bonâ-fide* and giving a certificate after ample observation, in regard to his own patient, and for his patient's benefit. But "meddling" is another affair, and in view of the fact that these certificates are not necessary, and are certainly not statutory, such meddling for a fee might easily be made to appear like malice. Of course in regard to the certificates required by law for the removal of a lunatic the matter is different, but even as to them, while the doctor who certifies for his own patient is fairly safe, the man who meddles, who goes out of his way, and fails to show that he has had ample opportunities for forming a proper judgment, runs many risks.

